# Improving Bacterial Metagenomic Research through Long-Read Sequencing

**DOI:** 10.3390/microorganisms12050935

**Published:** 2024-05-04

**Authors:** Noah Greenman, Sayf Al-Deen Hassouneh, Latifa S. Abdelli, Catherine Johnston, Taj Azarian

**Affiliations:** 1 College of Medicine, University of Central Florida, Orlando, FL 32827, USA; noah.greenman@ucf.edu (N.G.); sayfal-deen.hassouneh@ucf.edu (S.A.-D.H.); catherine.johnston@ucf.edu (C.J.); 2 Department of Health Science, College of Health Professions and Sciences, University of Central Florida, Orlando, FL 32816, USA; latifa.abdelli@ucf.edu

**Keywords:** metagenomics, shotgun metagenomic sequencing, next-generation sequencing, third-generation sequencing, taxonomic classification, metagenome-assembled genomes, simulation

## Abstract

Metagenomic sequencing analysis is central to investigating microbial communities in clinical and environmental studies. Short-read sequencing remains the primary approach for metagenomic research; however, long-read sequencing may offer advantages of improved metagenomic assembly and resolved taxonomic identification. To compare the relative performance for metagenomic studies, we simulated short- and long-read datasets using increasingly complex metagenomes comprising 10, 20, and 50 microbial taxa. Additionally, we used an empirical dataset of paired short- and long-read data generated from mouse fecal pellets to assess real-world performance. We compared metagenomic assembly quality, taxonomic classification, and metagenome-assembled genome (MAG) recovery rates. We show that long-read sequencing data significantly improve taxonomic classification and assembly quality. Metagenomic assemblies using simulated long reads were more complete and more contiguous with higher rates of MAG recovery. This resulted in more precise taxonomic classifications. Principal component analysis of empirical data demonstrated that sequencing technology affects compositional results as samples clustered by sequence type, not sample type. Overall, we highlight strengths of long-read metagenomic sequencing for microbiome studies, including improving the accuracy of classification and relative abundance estimates. These results will aid researchers when considering which sequencing approaches to use for metagenomic projects.

## 1. Introduction

Metagenomics has enabled the study of microbial communities for agricultural, ecological, and clinical applications without the need for isolation or lab cultivation of bacteria [1,2,3,4]. Advancement in the field has been driven by the continued improvement of sequencing technologies, which have enabled the generation of high-quality and low-cost genomic data for studying microbial populations with greater resolution [5]. Currently, the most widely used next-generation sequencing (NGS) technology is short-read sequencers, such as those from Illumina™ (Illumina, San Diego, CA, USA). Short-read sequencing platforms generate 75–300 bp single or paired-end reads with a per-base accuracy estimated at 99.9% [6]. Before the recent reduction in cost attributed to the widespread availability of NGS technology, so-called microbiome studies often employed metabarcoding, where small portions of the 16S rRNA gene are sequenced for taxonomic classification [7,8,9]. Although effective for genus-level classification, metabarcoding provides limited species-level resolution [10]. Where metabarcoding uses small regions of a single gene, shotgun metagenomics involves the sequencing of whole genome fragments, which can be used to reconstruct partial or whole microbial genomes through reference-based or de novo assembly approaches. Offering greater taxonomic resolution, de novo assembly is particularly useful for the identification of species that are non-culturable or lack robust representation in genomic databases. Shotgun metagenomic sequencing can also characterize variation in gene content, single-nucleotide polymorphisms, and mobile genetic elements [11,12].

In recent years, third-generation “long-read” sequencing platforms such as those from Oxford Nanopore Technologies™ (Oxford Nanopore Technologies, Oxford, UK) (ONT) and PacBio have increasingly been applied to clinical and biomedical research [11,13]. These platforms use a single-molecule sequencing approach that generates significantly longer DNA sequences [14]. A major strength of third-generation sequencers is their ability to span regions of genomes that are difficult for short-read sequencers to resolve, including common repetitive elements and other low-complexity regions [15]. Functional analyses are further improved with long reads as they can capture whole genes and preserve genetic structures [16]. Although there are numerous benefits, some notable drawbacks of long-read sequencing include a lower per-base accuracy. However, recent versions promise improvements in accuracy up to 99% [17,18]. In addition, available tools for long-read data analysis are relatively limited when compared to tools for the analysis of short-read data. More recently, the availability of tools has improved, with the number of long-read sequencing programs more than quadrupling since 2018 [19,20].

Due to its high accuracy, wide-spread availability, and well-established published pipelines, short-read sequencing is typically employed over long-read sequencing for metagenomic studies [21]. However, the advantages afforded by long-read sequencing make it particularly well-suited for metagenomic research. In this study, we sought to investigate the relative performance of short- and long-read sequencing for metagenomic analysis. To this end, we simulated metagenomic data from synthetic microbial communities of increasing complexity and measured precision, recall, and F-scores. We then applied paired short- and long-read sequencing in parallel to mouse fecal samples and empirically compared the relative performance.

## 2. Materials and Methods

### 2.1. Simulated Metagenomes of Synthetic Microbial Communities

**Figure 1:**
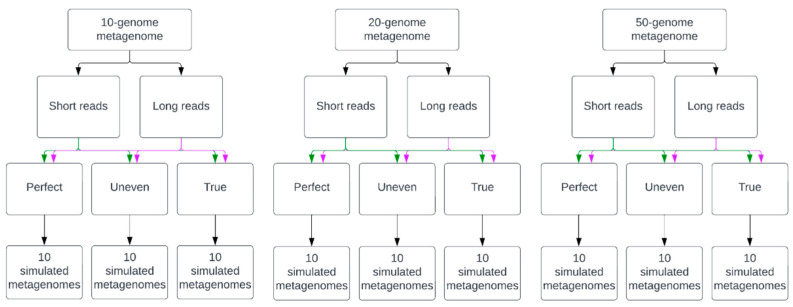
Simulation of metagenomic datasets of increasing complexity. “Perfect” metagenomic samples were simulated with no sequencing errors and an even abundance distribution of organisms. “Uneven” samples had no sequencing errors and randomly assigned abundance values for organisms in the metagenome, and “True” datasets had sequencing errors specific to each read type based on the platform they would be generated from and randomly assigned abundance values for members of each metagenome. Green arrows represent short-read data and purple arrows represent long-read data.

Nine datasets of simulated metagenomic sequencing reads were generated (Figure 1). Each set consisted of 10 unique, simulated metagenomic samples of varying complexity. Complexity was defined by the number of taxa, the relative abundance of organisms in a metagenome, and the presence or absence of simulated sequencing errors. The size of a simulated metagenome consisted of genomes from 10, 20, or 50 taxa with abundances either evenly distributed or randomly generated and simulated sequencing errors present or absent. The three types of sequence data included “Perfect”, “Uneven”, and “True” datasets. Perfect datasets consisted of reads with no errors and even organism abundance distribution, Uneven datasets without errors and random abundance values for each organism, and True datasets with simulated errors specific to each sequencing platform for a given sequence type and random abundance values for each organism. Simulated long reads were generated to reflect sequence data from ONT’s MinION™ (Oxford Nanopore Technologies, Oxford, UK) and short reads mirrored sequence data from Illumina’s™ HiSeq™ sequencer (Illumina, San Diego, CA, USA). 

Construction of synthetic metagenomes was performed using an assembly structure report downloaded from NCBI (downloaded 04/18/2022; Figure S1). This report contained all bacteria present in RefSeq with the following criteria for inclusion: (1) genomes must be part of the latest RefSeq database, (2) they must have a complete genome assembly, and (3) all anomalous assemblies are excluded. 

Simulation of ONT sequence data was performed using NanoSim version 3.0.0 [22]. NanoSim input files (genome, abundance, and DNA lists) were created using a custom python script specifying parameters according to the level of metagenome complexity. The genome list (-gl) contained randomly selected taxa from the assembly structure report. The number of taxa selected was based on the desired size of the metagenome: 10, 20, or 50. Similar organisms could be present in a metagenome, such as different strains of the same species. The abundance list (-al) contained each organism and a value defining the abundance of that organism in the metagenome. This value was either equal across all organisms or randomly generated such that the total abundance of all organisms was 100%. The DNA list (-dl) contained all genetic molecules present in each organism’s genome. This included an organism’s chromosome and any plasmids, as well as whether the molecule was circular or linear. For simulating errors (-c), the pre-trained model ‘metagenome_ERR3152364_Even’ supplied by NanoSim was used. The basecaller (-b) specified was guppy. The ‘--perfect’ option was specified for datasets without simulated errors, including “Perfect” and “Uneven” datasets. This option was not used for “True” datasets. All simulated reads were generated in FASTQ format (--fastq). The total number of reads generated per metagenome was 650,000, with reads having an N50 of approximately 5.2 Kbps. Total bases for each sample were approximately 2.67 Gbps. These values were selected to mimic realistic outputs from nanopore sequencing. For long reads, filtering was performed using Filtlong version 0.2.1 [23]. Long reads were filtered by a minimum read length of 1000 bp (--min_length 1000).

Simulation of short-read sequence data from an Illumina™ HighSeq™ was conducted using the randomreads.sh script from BBMap version 37.93 [24]. Ten million paired-end reads were generated for each simulated metagenome. When creating metagenomic short-read data, the abundance of each genome was determined from the abundance list used for simulating long reads. Dividing 10 million by the abundance for each genome yielded the number of short reads to simulate. Each iteration of randomreads.sh took a single genome and the number of reads specific to that genome and output a FASTQ file containing interleaved paired-end short reads. All datasets consisted of paired-end reads that were 150 bp in length (paired = t, len = 150), named according to their genomic origin and without coordinates (simplenames = t); the read quality was set to 36 (q = 36), and a single insert size distribution was specified (superflat = t). Error rates were controlled by specifying the maximum number of SNPs (maxsnps), insertions (maxinss), insertion rate (insrate), deletions (maxdels), deletion rate (delrate), substitutions (maxsubs), and substitution rate (subrate). For “Perfect” and “Uneven” datasets, all values for controlling errors were set to 0 and an additional argument controlling the inclusion of errors was set to false (adderrors = f). “True” datasets used the following values for error-controlling parameters: maxsnps = 3, maxinss = 2, maxdels = 2, maxsubs = 2, insrate = 0.00000315, delrate = 0.000005, subrate = 0.0033, and adderrors = t. The chosen error rates are based on average error rates observed by Schrimer and colleagues in their paper on Illumina™ error profiles from metagenomic data [25]. Simulated reads for each genome of a metagenome were concatenated. Then, to induce random ordering of reads, the tool shuffle.sh from BBMap was used with default parameters. Read pairs were then separated into two FASTQ files using BBMap’s reformat.sh tool, specifying that the single, shuffled, interleaved file be split into R1 and R2 FASTQ files. No filtering was performed on short reads as all reads possessed a q-score of 36.

### 2.2. Metagenome Assembly and Quality Assessment

Long-read assembly was performed using Flye version 2.9-b1768 [26]. Default parameters for Flye were used with the addition of ‘--nano-raw’ and ‘--meta’ to denote assembly of uncorrected reads from a metagenomic sample. SPAdes version 3.15.5 was used for metagenomic assembly of simulated short-read data [27]. Default options for SPAdes were used except for the following: ‘--meta’ was specified to indicate that the sequence data were from a metagenomic sample; kmer sizes (-k) consisted of a list of integers: 21, 33, 55, 77, 99, and 127; ‘--phred-offset’ was set to 33 due to simulated short reads not possessing defined signatures of the PHRED quality; and ‘--only assembler’ was specified to disable read error correction. Metagenomic assembly quality was evaluated using metaQUAST version 5.0.2 [28]. Default parameters were used except for supplying the directory containing the reference genomes for each metagenome to ‘-r’. 

### 2.3. Metagenome-Assembled Genome Recovery and Taxonomic Classification

Metagenome-assembled genomes (MAGs) were generated using the following steps. First, long reads were mapped back to the assemblies using minimap2 version 2.24-r1122. For long reads, the flag ‘-x’ was given the ‘map-ont’ argument to optimize alignment parameters for nanopore sequence data [29]. Short-read headers were shortened using the tool seqtk-1.4 version r122’s ‘rename’ function [30]. Short reads were then mapped using bwa-mem2 version 2.2.1 by first creating an index from a metagenome’s assembly file using ‘bwa-mem2 index’, and then mapping the reads back using ‘bwa-mem2 mem’. Each alignment process produced an unsorted SAM file which was then converted into sorted BAM files using Samtools version 1.16.1 with options ‘samtools view -b’ for creating the BAM file, and ‘samtools sort’ for sorting the BAM file [31]. A coverage file was generated for each read type using CoverM version 0.6.1 using the default ‘-m mean’ for coverage estimation [32]. For long reads, the mapper option for CoverM was set to ‘-p minimap2-ont’, and for short reads it was ‘-p bwa-mem’. Each coverage file was used to bin each read type’s respective assemblies using MetaBAT 2 version 2.15 with default parameters except for the addition of the ‘--cvExt’ argument to denote coverage files that originated from a third-party tool [33]. Binned contigs were then assessed for completeness and contamination using CheckM2 version 1.0.1 with default parameters [34]. Taxonomic classification was performed using Kraken2 version 2.1.2 for both short- and long-read metagenomic assemblies [35]. Default parameters were used with the ‘--use-names’ flag included. The kraken2 report and output files were kept for further analysis. 

### 2.4. Result Evaluation

For visual assessment of metagenome assembly quality, assembly graphs were visualized using Bandage version 0.8.1 [36]. Resulting values for genome fraction recovery, NGA50, and number of misassemblies from metaQUAST were analyzed using the *statannotations* python package [37]. Statistical significance was evaluated using a two-sided Mann–Whitney U test with Bonferroni correction. A *p*-value threshold of 0.05 was used to determine significance.

Performance evaluation of taxonomic classification from reads and assemblies was accomplished by calculating precision, recall, and F-scores. Precision was defined as the rate of successful taxonomic assignment of a read/contig to the correct taxon within the metagenome over the total number of taxonomic assignments. Also referred to as the false positive rate, the equation used was $Precision=\frac{TP}{\left(TP+FP\right)}$, where TP is the true positive rate and FP is the false positive rate, or an incorrect classification of a read/contig at a given taxonomic rank. Recall, also defined as the rate of false negatives, was determined using the equation $Recall=\frac{TP}{total}$, where ‘total’ is the expected number of distinct organisms at a specified taxonomic rank. F-score, or the harmonic mean, generates an overall score factoring in both precision and recall. F-score was calculated using the equation $F1=\frac{2*\left(precision*recall\right)}{\left(precision+recall\right)}$. A statistical comparison of precision, recall, and F-score was performed using a one-way ANOVA for assemblies and a Mann–Whitney U test with Bonferroni correction for reads with the statannotations python package. A *p*-value threshold of 0.05 was used to determine significance. 

An estimation of the relative abundance of taxa at the genus and species level was carried out using Bracken version 2.8 [38]. Short and long reads were processed through Kraken2 to generate report files that were then used as inputs for Bracken. Bracken then returned a report file containing estimates of relative abundance. For each genome in a metagenome, the predicted abundance was plotted against the actual abundance in a scatterplot along with a linear regression line using a linear regression function from SciPy version 1.10.1 [39]. R-values, slope, and *p*-values for each read type’s line were reported. 

MAG recovery rates were determined by comparing the total number of MAGs recovered by the binning of contigs from short- and long-read metagenomic assemblies. A MAG’s quality is determined by the completeness and contamination values of contigs assigned to a bin by MetaBAT2 and assessed by CheckM2. CheckM2 defines completeness as the percentage of universal, bacteria-specific gene markers present in a given bin. Contamination for CheckM2 is based on how many of these markers exist in multiple copies, which indicates the presence of contigs that do not belong in a particular bin. For the minimum threshold of a bin to be considered an MAG, the cutoffs were defined as a completeness ≥50% and contamination ≤10% [40]. For each of the 9 sets of metagenomes, the average number of MAGs recovered from short- and long-read assemblies was recorded. A Mann–Whitney U Test with Bonferroni correction was applied using the *statannotations* python package to compare differences in MAG recovery rates between short- and long-read assemblies. A *p*-value threshold of 0.05 was used to determine significance. Bins determined to be MAGs were also evaluated on quality, which was defined by completeness only. High-quality MAGs had a completeness ≥90%, medium-quality MAGs had a completeness ≥70% and <90%, and low-quality MAGs had a completeness ≥50% and <70%.

### 2.5. Comparison of Experimental Metagenomic Data

To examine whether read type affects the assessment of microbial composition, we generated short- and long-read sequencing data from 19 mouse fecal pellets collected during ongoing experimental studies related to the impact of diet on the gut microbiome. In particular, we investigated how the bacterial metabolite propionic acid (PPA) changes the composition of the gut microbiome among mice progeny who were fed PPA-rich diets. More generally, feces are rich in microbial DNA while having low concentrations of host DNA, providing an ideal sample type for compositional experiments [41]. Long-read sequencing data were generated using the ONT GridION. Prior to DNA extraction, host DNA was depleted using an enzymatic approach as described previously with some modifications [13]. Each sample extraction used 1–2 mouse fecal pellets. The fecal pellets were homogenized in 1 mL of InhibitEX buffer (Cat. No./ID: 19593, Qiagen, Hilden, Germany). After pelleting the homogenate and aspirating off the liquid, 250 µL of 1X PBS and 250 µL of 4.4% saponin was added and mixed thoroughly before resting at room temperature for 10 min. Nuclease-free water (350 µL) along with 12 µL of 5M NaCl were added to induce osmotic lysis. After pelleting, the liquid was again aspirated off and the pellet was resuspended in 100 µL of 1X PBS. One-hundred microliters of HL-SAN enzyme buffer was added (5.5 M NaCl and 100 mM MgCl2 in nuclease-free water), followed by 10 µL of HL-SAN endonuclease (Article No. 70910-202, ArcticZymes, Tromsø, Norway) before being incubated in a shaking incubator at 37 °C for 30 min at 1000 rpm. Two final washes of the pellet were performed using 800 and 1000 µL of 1X PBS before proceeding with extraction. Genomic DNA (gDNA) was extracted and purified using the New England Biolabs Monarch^®^ Genomic DNA Purification Kit (New England Biolabs, Ipswich, MA, USA) following the manufacturer’s instructions. The quality and concentration of gDNA were assessed using an Agilent 4200 TapeStation System and a Qubit 4 Fluorometer. Sequencing libraries for nanopore sequencing were prepared using the Rapid PCR Barcoding Kit (SQK-RPB004). Using an Oxford Nanopore GridION™ (Oxford Nanopore Technologies, Oxford, UK), libraries were sequenced on an R9.4.1 flow cell generating an average of 1.84 Gbps of sequence data with a mean read length of 3491.62 bps and a mean read quality of 12.9. The longest read produced was 42,266 bps. Short-read sequencing was performed by SeqCenter LLC (SeqCenter, Pittsburgh, PA, USA) using an Illumina™ NovaSeq™ with a Nextera Flex Library Preparation Kit^®^ (Illumina, San Diego, CA, USA) and V3 flow cell chemistry to produce an average of 2.53 Gbps of paired-end 2 × 150 bp reads across all samples. Illumina data were filtered using Trimmomatic v0.39 for paired-end reads with the following trimming settings: SLIDINGWINDOW:4:20 MINLEN:100.

Analysis followed the methodological approaches described previously for short and long reads, respectively. Reads were first classified using Kraken2 and relative abundances were estimated using Bracken. Before analysis, Bracken reports were trimmed to remove all taxonomic identifications where the number of reads assigned to an organism fell below a given threshold of 1/20,000 (0.00005). To our knowledge, no studies using shotgun metagenomic sequencing have given the expected taxonomic diversity of the mouse gut microbiome at the genus or species level. The Murine Microbiome Database consists of 1732 genera and 4703 species; however, these data come from 16S sequence analysis [42]. An alternative database, the Comprehensive Mouse Microbiota Genome (CMMG), derived 1573 species from mouse gut metagenomes combined with known reference metagenome-assembled genomes at the time of its creation [43]. In this study, we empirically defined a cutoff where the count of unique taxa was closer to that of the CMMG, as 16S sequencing often reports higher diversity. Initial plotting of the number of unique taxa showed counts of over 1750 genera and over 6000 species for both short- and long-read data when no abundance cutoff was applied. Using a cutoff of 1/20,000 yielded unique taxa counts for species similar to that of the CMMG. Short- and long-read data for a group were then compared to each other using the beta_diversity.py script from the KrakenTools suite [44]. Qualitative assessment was performed by generating a hierarchically clustered heatmap of the resulting Bray–Curtis Dissimilarity matrix. For quantitative analysis, a principal component analysis (PCA) was performed. First, data were transformed using a center-log ratio transformation [45]. Then, PCA was run using the PCA function from scikit-learn’s decomposition library [46]. Briefly, sample IDs were separated as features and abundance values for each taxon at the genus and species level were retained. Abundance values for unique taxa were then given to the PCA object which created a fit and subsequently applied dimensional reduction to the abundance data. The resulting principal components were kept in a table from which the top two principal components were plotted. 

## 3. Results

### 3.1. Comparison of Metagenomic Assembly Completeness

Metagenomic assemblies for each synthetic dataset were qualitatively assessed through visualization (Figure S1). Long-read assemblies produced graphs with greater contiguity and often resulted in the circularization of unitigs, an indication that they represented complete chromosomes or, in the case of smaller, circularized contigs, plasmids.

**Figure 2:**
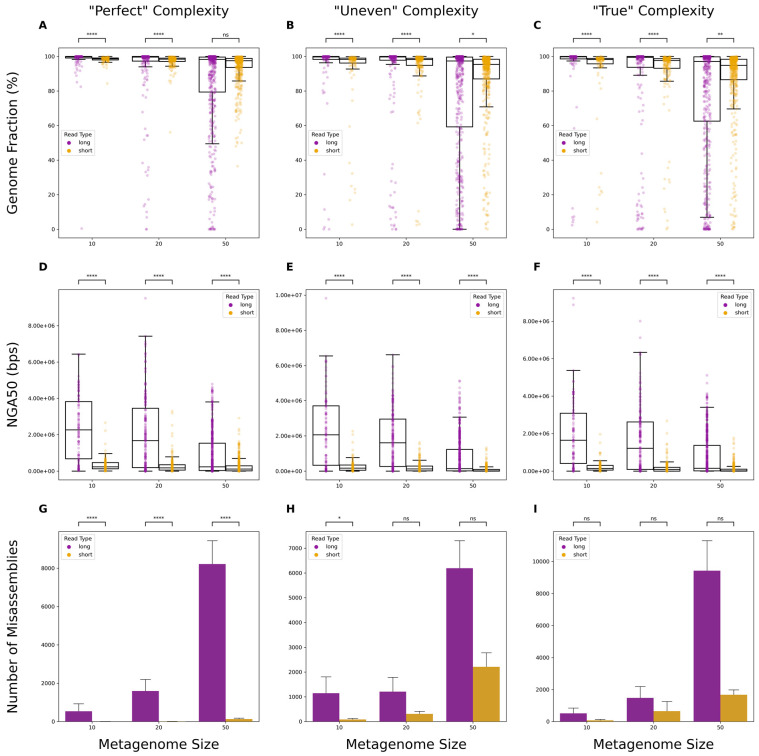
Comparisons of assembly quality metrics. Boxplots depicting differences in genome fraction values (**A**–**C**) and NGA50 (**D**–**F**) from all genomes in a set of metagenomes. Bar plots depicting total misassembly counts across all genomes in a set of metagenomes (**G**–**I**). “Perfect” denotes that simulated data have no errors and abundances are evenly distributed for each organism in the metagenome. “Uneven” denotes that simulated data have no errors but variable abundances for each organism. “True” denotes that simulated data have simulated sequencing errors based on the read type, as well as variable abundances for each organism. Significance was calculated using a Mann–Whitney U Test with Bonferroni correction. ns: non-significant *p*-value (*p* > 0.05), *: *p* ≤ 0.05, **: *p* ≤ 0.01, ****: *p* ≤ 0.0001.

Assemblies from long reads demonstrated significantly higher genome fraction coverage than their short-read counterparts (Figure 2A–C; Supplementary Table S1). This was observed across all metagenome sets, regardless of complexity, except for the synthetic dataset composed of 50 organisms with perfect reads (Figure 2A). Similar results were observed when comparing assemblies by NGA50 values, with long-read data showing significantly higher scores (Figure 2D–F; Supplementary Table S1). With “Perfect” data, short reads produced significantly fewer misassemblies; however, this difference was not significant for larger “Uneven” and all “True” datasets (Figure 2G–I; Supplementary Table S1). 

### 3.2. Evaluating Taxonomic Classification

**Figure 3:**
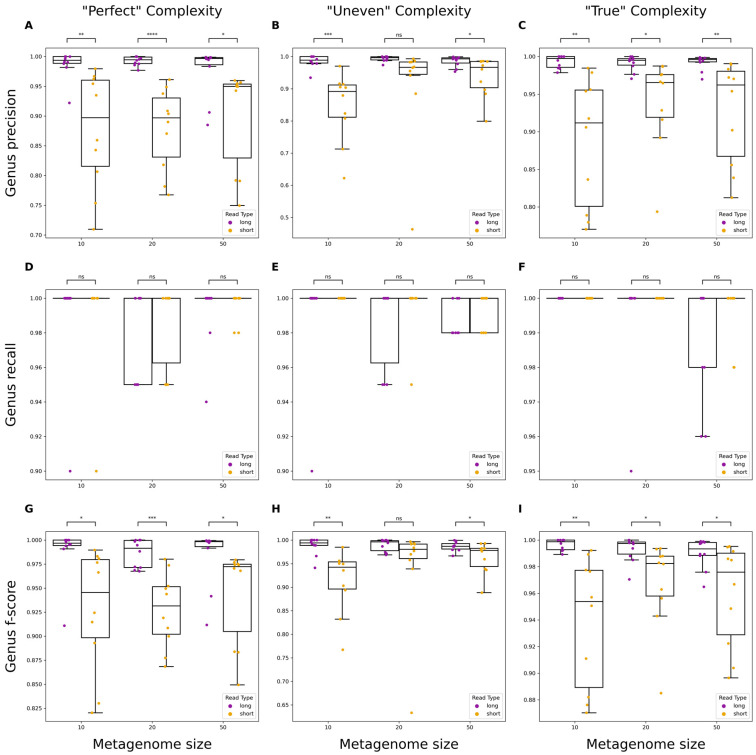
Performance evaluation of genus-level classification on metagenomic assemblies using short or long reads. Boxplots showing precision, recall, and F-score metrics for metagenomes of varying size and complexity. “Perfect” simulated reads have no errors and even abundances across organisms (**A**,**D**,**G**), “Uneven” have no errors and randomly varied abundances across organisms (**B**,**E**,**H**), and “True” have simulated errors specific to each read type and varied abundances across organisms (**C**,**F**,**I**). Significance was calculated using a one-way ANOVA. ns: non-significant *p*-value (*p* > 0.05; blank means *p* = 1), *: *p* ≤ 0.05, **: *p* ≤ 0.01, ***: *p* ≤ 0.001, ****: *p* ≤ 0.0001.

**Figure 4:**
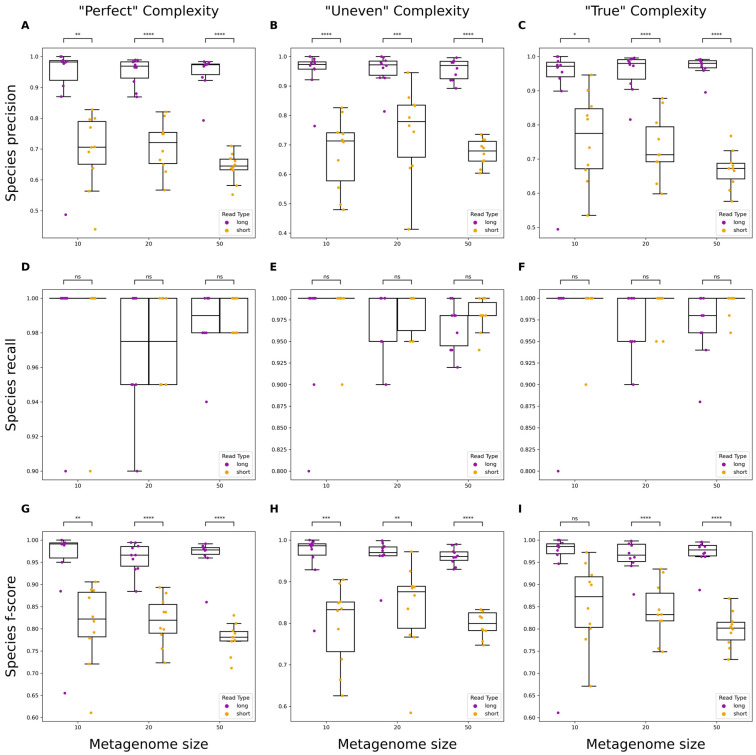
Performance evaluation of species-level classification of metagenomic assemblies using short or long reads. Boxplots showing precision, recall, and F-score metrics for metagenomes of varying size and complexity. “Perfect” simulated reads have no errors and even abundances across organisms (**A**,**D**,**G**), “Uneven” have no errors and randomly varied abundances across organisms (**B**,**E**,**H**), and “True” have simulated errors specific to each read type and varied abundances across organisms (**C**,**F**,**I**). Significance was calculated using a one-way ANOVA. ns: non-significant *p*-value (*p* > 0.05; blank means *p* = 1), *: *p* ≤ 0.05, **: *p* ≤ 0.01, ***: *p* ≤ 0.001, ****: *p* ≤ 0.0001.

Evaluation of taxonomic classifications using assemblies at the genus and species levels was assessed for precision, recall, and F-score. Neither read types significantly differed when measuring the recall of taxa (Figure 3D–F and Figure 4D–F). However, long reads significantly outperformed short reads in precision across almost all levels of metagenome complexity at both the genus and species levels (Figure 3A–C and Figure 4A–C). The F-score, or the harmonic mean of the precision and recall, was calculated to measure the accuracy of classification results using either short or long reads. Long reads demonstrated significantly higher F-scores than short reads across almost all levels of complexity, indicating that long reads provide greater accuracy for taxonomic classification.

### 3.3. Estimating Relative Abundance Using Short- and Long-Read Data

**Figure 5:**
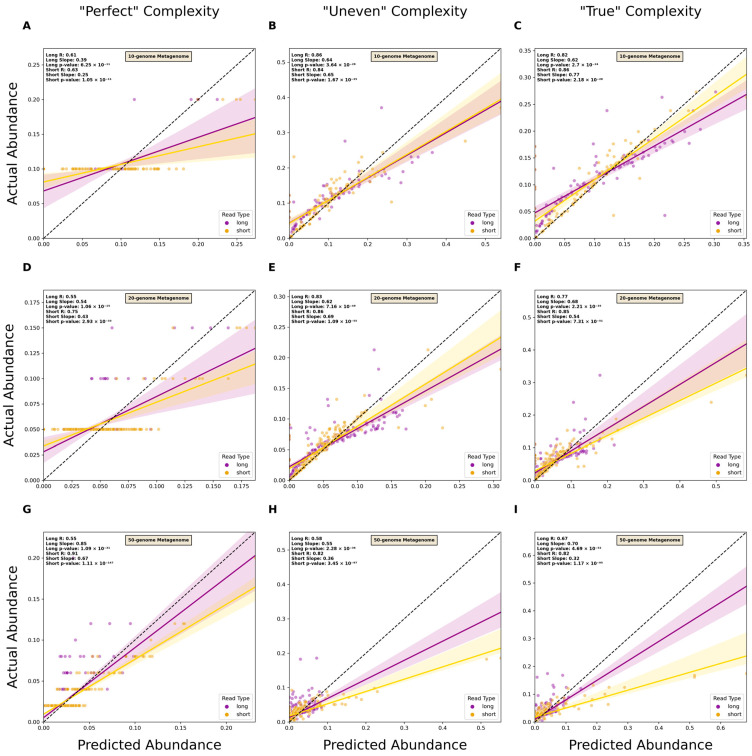
Comparison of short- and long-read capacity for species-level relative abundance estimation. Scatterplots of predicted versus actual abundance values for genomes present in simulated metagenomes from short- and long-read data. Read types were compared across metagenomes of varying complexity at both the genus and species levels. “Perfect” datasets consisted of reads without sequencing errors and in which organism abundance was evenly distributed (**A**,**D**,**G**), “Uneven” consisted of reads without sequencing errors and randomly distributed abundances of organisms (**B**,**E**,**H**), and “True” consisted of reads with simulated errors and randomly distributed abundances of organisms (**C**,**F**,**I**). A linear regression line was plotted for each read type. Each line has its reported R-value, slope, and *p*-value. The dotted line represents the 1-to-1 line where values of predicted abundance match values of actual abundance.

Before analyzing the performance of each read type when used for relative abundance estimation, the classification capabilities of the reads were first assessed. The precision, recall, and F-score of genus-level classification were not significantly affected by read type in most cases, which differed from classification using metagenomic assemblies (Supplementary Figure S2). However, the performance for each read type on species-level classification was significantly improved when using long reads (Supplementary Figure S3). Both precision and F-score were higher for classifications using long reads and recall was not significantly different between the two. Examining accuracy in the estimation of relative abundance found that long reads outperformed short reads in most cases at the genus and species levels (Figure 5 and Figure S4 ). 

### 3.4. Metagenome-Assembled Genome (MAG) Recovery from Short- and Long-Read Data

**Figure 6:**
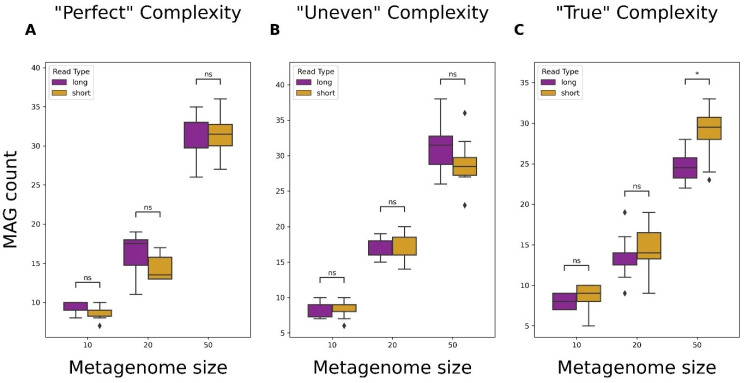
Comparison of metagenome-assembled genome (MAG) recovery capabilities from assemblies produced using short or long reads. Total number of MAGs recovered from assemblies of simulated short- or long-read sequence data. “Perfect” datasets consisted of reads without sequencing errors and organism abundance being evenly distributed (**A**), “Uneven” consisted of reads without sequencing errors and organism abundance being randomly assigned (**B**), and “True” consisted of reads with simulated sequencing errors and organism abundance being randomly assigned (**C**). Significance was calculated using a Mann–Whitney U Test with Bonferroni correction. ns: non-significant *p*-value (*p* > 0.05), *: *p* ≤ 0.05.

In most cases, neither read type resulted in more MAGs being recovered except for short reads producing more MAGs from “True” 50-genome metagenomes (Figure 6C). It was found that the binning of contigs from long-read assemblies generally produced more total bins than assemblies produced from short reads (Supplementary Table S2). Following this observation, the quality of MAGs recovered was evaluated. Bins were assigned a quality of high, medium, or low, depending on their completeness. Long reads did not result in more high-quality MAGs at any level of complexity; however, they did show significantly more medium- and low-quality MAGs in some cases (Supplementary Figure S5D,G,H). Complementing Figure 6, short reads recovered significantly high-quality MAGs from the most complex datasets (Supplementary Figure S5I).

### 3.5. Microbial Compositional Differences between Short and Long Reads

**Figure 7:**
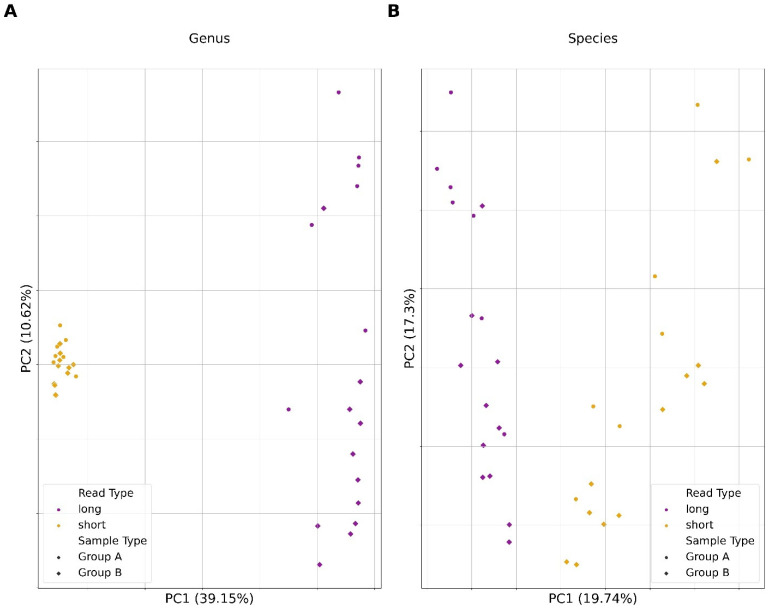
Principal component analysis (PCA) results from short- and long-read sequence data analysis of empirical metagenomic samples from mouse fecal pellets (**A**,**B**). PCA plot depicting the distribution of metagenomic samples according to their two main principal components. Coloration indicates the read type: purple for long-read data and yellow for short-read data. Shape indicates the sample type. Principal components 1 and 2 are on the *x* and *y* axis, respectively, with their explained variance ratios written as a percentage.

Using experimental data obtained from mouse fecal pellets, we examined whether read type significantly altered inferences of microbial composition for a metagenomic sample. Qualitative assessment showed clustering of results around similar read types instead of around sample types (Figure S6). This trend was also observed when results were run through a PCA, where samples clustered around similar read types instead of around similar sample types (Figure 7).

## 4. Discussion

For metagenomic studies of microbial communities, long-read sequencing platforms potentially offer numerous advantages, yet short-read sequencing remains the most commonly applied technology [47]. We sought to quantitatively assess the use of long-read sequence data by using simulated metagenomes with increasing complexity. Evaluation of read type performance across several metrics showed that long and short reads possess respective strengths. We also used paired long- and short-read metagenomic data from murine fecal samples to assess the generalizability of our findings. Overall, we highlight that the choice of sequence type has a direct impact on the ability to accurately assemble metagenomes, identify members of a diverse microbial community, and recover MAGs from metagenomic sequence data. 

For optimal performance, sequencing data must be capable of producing high-quality assemblies for taxonomic classification, relative abundance estimation, functional annotation, and other related analyses. We show that the simulated long-read assemblies had significantly higher-percentage genome coverages than short-read assemblies. Analyses that rely on examining contiguous sequences, such as gene annotation, would thus benefit from assemblies generated using long-read data. Short reads are known to struggle with repetitive regions that are common in many bacterial genomes [48], which likely explains the lower genome coverages and greater discontiguity observed from short-read assemblies. Higher NGA50 values indicate greater contiguity, further supporting the findings highlighted by the higher genome fraction observed. Regarding misassembly rates, short reads did produce fewer misassemblies in all “Perfect” datasets (Figure 2G). However, in more complex datasets, the observed differences were found to not be significant in most cases (Figure 2H,I). One explanation is that metagenomic assemblers attempt to balance accuracy and contiguity, thus increasing the possibility of misassemblies [26]. The lower per-base accuracy of long-read sequencers has long been a reason why researchers tend to favor short reads. Our results highlight the shrinking gap in result quality of assemblies produced with either short or long reads.

Taxonomic classification is central to metagenomic studies and several tools exist for use on filtered reads or assembled contigs. Previous studies have compared classification using partial and full 16S sequencing using short- and long-read platforms, respectively [49]. Here, we demonstrate the capabilities of each read type when classifying whole metagenomic sequence data. For long-read assemblies, we found that classifications demonstrated significantly higher precision at both the genus and species levels overall. The greater precision likely stems from long-read assemblies being more contiguous, thus having fewer small fragments that could be improperly classified like in short-read assemblies. When classifying reads at the genus level, both long and short reads performed similarly (Figure S2). This was partly explained by long reads having a lower precision compared to classifications with contigs. This is expected, given that there are more reads than contigs and thus more chances for reads to be misassigned to an improper taxon. When analyzing reads at a finer taxonomic resolution, long reads performed significantly better. For differentiation of species within a genus, larger reads provide more information for distinguishing the genome of origin. For assemblies and filtered reads, we observed no difference in sensitivity. These findings are relevant for the potential application of long-read metagenomic sequencing to infectious disease diagnostics [50,51,52]. For example, the ability of ONT sequencing platforms to generate sequencing reads in real time, which can be used for taxonomic classification without sacrificing precision or sensitivity, is invaluable and avoids lengthy culturing, isolation, and molecular diagnostics.

In addition to taxonomic classification, assessment of differences in the relative abundance of species is of great interest. This generally involves the alignment of reads to a reference genome database and a subsequent estimation of abundances from the coverage, which can be performed using both short and long reads [53,54]. Our results showed that predicted abundances from long-read data were more likely to be close to the actual abundance data for both genus- and species-level classification (Figure 5 and Figure S4). This was likely due to the role of Kraken2’s results in Bracken’s estimation of organismal relative abundances. Bracken takes the initial classifications and then attempts to re-assign reads to proper taxa using a Bayesian-based approach. Having greater accuracy in these classifications enables Bracken to be more likely to assign reads to taxa accurately. Assessment of relative abundance thus benefitted from the higher precision of long reads. Research that derives conclusions based on significant shifts in abundances of all taxa or specific organisms can thus benefit from reliance on abundance estimation. Also, in clinical diagnostics, accurate estimation of abundance may enable clinicians to identify the etiologic microbe associated with the disease of interest [55]. 

A considerable challenge in microbial research is that most bacteria are non-culturable, which makes the identification of organisms of potential importance difficult. Using shotgun sequencing and de novo metagenomic assembly, it is possible to recover both known and novel genomes and infer the identity and functional capacities of the organisms [56]. Assemblies produced from either read type resulted in similar MAG recovery rates, except short reads did identify more MAGs in “True” 50-genome metagenomes (Figure 6). A key tool in the binning process was MetaBAT2, which is highly dependent on assembly quality for performance. As a result, while long reads were more contiguous and often more complete, misassembly rates may have impacted read alignment during the MAG identification process. This is supported by our observation that long-read assemblies contained more misassembles, even though misassembly rates were not significantly different between read types (Supplementary Table S2). This likely resulted in more bins with fewer contigs spread among them and, therefore, lower completeness due to the cutoffs used for MAG reporting. This also explains why long reads produced more medium- or low-quality MAGs at times, since MAG quality was primarily determined by completeness; thus, having contigs inadequately binned would result in lower completeness of MAGs. 

The selection of sequencing platforms has previously been shown to directly impact the taxonomic assignment of partial/total 16S rRNA sequences [57]. In the present study, bacterial metagenomic DNA extracted from murine fecal samples was sequenced using both short- and long-read sequencing platforms. Qualitative assessment measuring the beta diversity among samples of the same type, but different sequence data, revealed samples clustered by sequence type, not sample type (Supplementary Figure S6). Comparisons using PCA revealed the same trend of samples clustered by sequence type rather than by sample type (Figure 7). This is likely due to an inflation of misclassified taxa in short-read assemblies, causing clustering to be associated with those unique taxa. This is consistent with our findings in that simulated short-read data produced more false positives than long reads. This, in turn, artificially increased the diversity of the sample and explains clustering due to read type instead of sample type. 

Taken together, our results show that long-read data offer certain advantages toward the accurate representation of a metagenome. Specifically, long reads resulted in greater accuracy when taxonomically classifying contigs and reads, and their assemblies were more contiguous and returned greater genome fractions for genomes within a metagenome. While this study provides evidence supporting the use of long-read sequencing for metagenomic research, it does have some limitations. The most complex metagenomes that were simulated consisted of 50 organisms with variable abundance and simulated sequencing errors specific to each read type, but empirical data may consist of hundreds or even thousands of unique organisms [48]. Future work could employ simulations that mimic high-diversity and high-abundance distributions observed in real metagenomic samples; however, we feel that increasing taxa would only increase the disparity between sequence data types. Another consideration is that pipelines for processing and analyzing metagenomic data can vary considerably. Various tools exist for read correction, assembly, polishing, taxonomic classification, and binning, all of which can affect results. Steps such as read error correction and assembly polishing are often used in metagenomic studies; however, we did not include them here to reduce computational requirements and to limit variables in the comparison, especially if they are unique to one read type. Another limitation was that binning algorithms are usually designed with short reads in mind, leveraging the higher coverage of data that accompanies short-read sequencing. In recent years, tools such as LRBinner have emerged, offering better binning for error-prone long reads [58], but those were not applied here. Overall, we aimed to use the most commonly employed tools used for these studies at the time of writing. Lastly, we used a limited number of real-world samples, which could be expanded in future studies.

Short- and long-read sequencing have their respective places in metagenomic research. Short-read sequencing does have its strengths, especially with pipelines for analyzing short-read data being more established. Our results highlight that both read types perform comparably well across several metagenomic analyses, particularly assembly quality and MAG recovery. For studies concerned with misassemblies, such as those focused on specific species of interest, short reads produce significantly fewer and may be preferable. For studies concerned with microbial composition, long reads provide higher precision with similar sensitivity to short reads despite lower sequencing depths. They also provide better estimations of organismal abundance within metagenomic samples. Leveraging the strengths of both read types is also possible. Tools such as OPERA-MS combine long reads containing more genetic information per read with high-accuracy short reads [59]. Other programs like PolyPolish enable the use of short reads for improving long-read assembly quality [60]. Further advancements may shift practice towards the use of hybrid approaches, as has occurred with genome sequencing. In conclusion, our results show that long-read sequence data outperform short read sequence data when used for taxonomic classification and metagenomic assembly contiguity. Further, this informs our future experimental studies on the impact of diet on the composition of the gut microbiome. This work provides evidence for the consideration of long-read sequencing for research focused on accurate reconstruction of microbial populations and its strength in clinical diagnostics. 

## Data Availability

The experimental metagenomic sequence data used for comparison of short- and long-read data from the same source are available from NCBI’s Sequence Read Archive (SRA) under Bioproject accession ID PRJNA1092431. The raw, simulated data supporting the conclusions of this article will be made available by the authors upon request.

## Supplementary Materials

The following supporting information can be downloaded at https://www.mdpi.com/article/10.3390/microorganisms12050935/s1: Figure S1: Supplemental Figure S1. Assembly graphs from a “True” 10-genome metagenome visualized by Bandage; Figure S2: Supplemental Figure S2. Performance evaluation of genus-level classification using short and long reads; Figure S3: Supplemental Figure S3. Performance evaluation of species-level classification using short and long reads; Figure S4. Comparison of short and long read capacity for genus-level relative abundance estimation; Figure S5: Comparison of metagenome-assembled genome (MAG) counts recovered from short and long read assemblies by quality; Figure S6: Hierarchically clustered heatmap of Bray-Curtis Dissimilarity; Table S1: Results of Mann-Whitney U testing for genome fraction, NGA50, and number of misassemblies; Table S2. Bin and metagenome-assembled genome (MAG) counts from metagenomic samples.
