# Supplementary material for: Improving Bacterial Metagenomic Research through Long-Read Sequencing
Source: Microorganisms. 2024 May 4;12(5):935. doi: 10.3390/microorganisms12050935 (PMC11124196; doi:10.3390/microorganisms12050935)
Supplement: Supplementary file 1 [file microorganisms-12-00935-s001.zip › microorganisms-2981447-supplementary.pdf]

microorganisms

MDPI

Article

Improving Bacterial Metagenomic Research through Long-Read Sequencing

Noah Greenman [1], Sayf Al-Deen Hassouneh [1], Latifa S. Abdelli [2], Catherine Johnston [1] and Taj Azarian [1,*]

Supplementary Materials

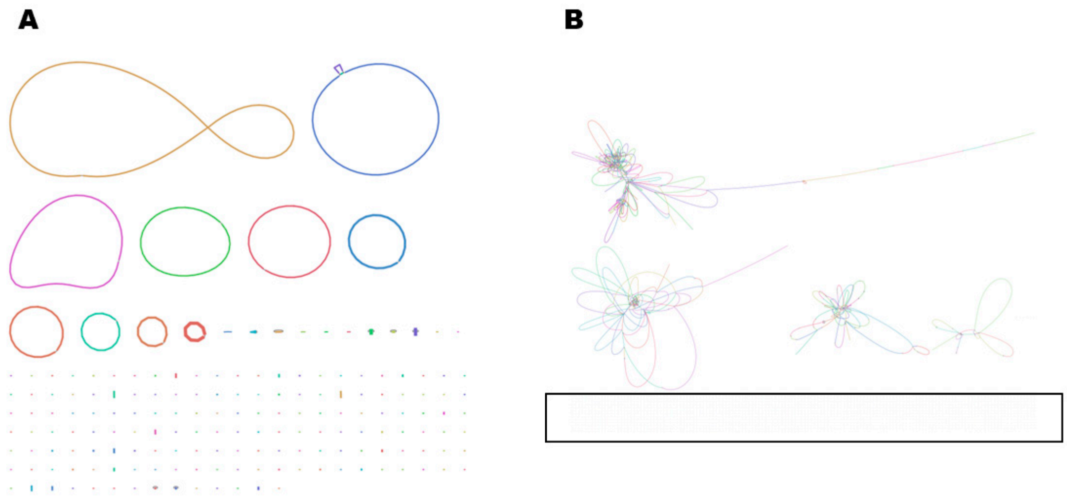

Supplemental Figure S1. Assembly graphs from a "True" 10-genome metagenome visualized by Bandage. (2A) Bandage graph generated from metagenomic assembly of long-read sequence data. (2B) Bandage graph generated from metagenomic assembly of short-read sequence data. The black box highlights small, disconnected contigs.

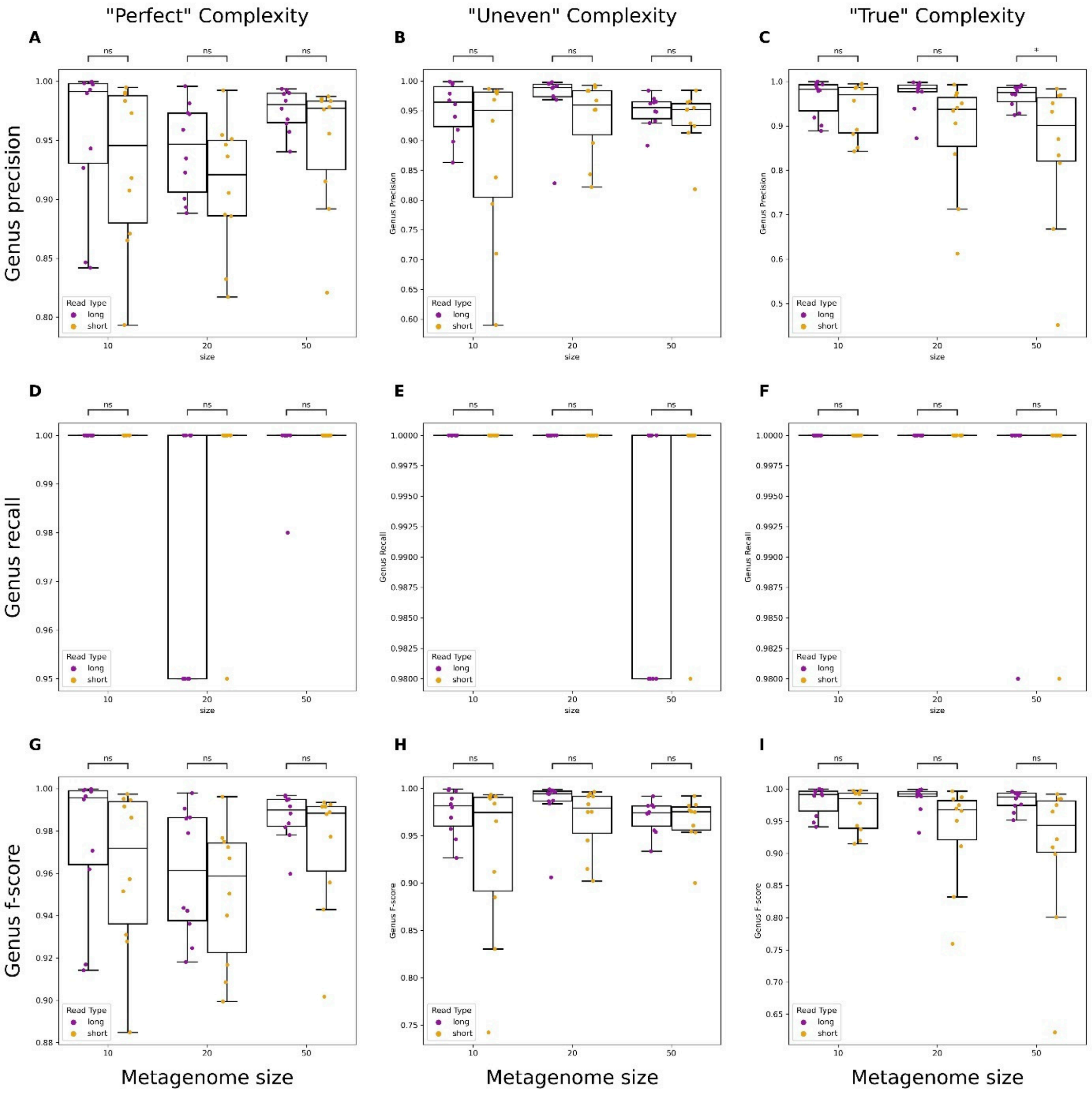

Supplemental Figure S2. Performance evaluation of genus-level classification using short and long reads. Boxplots showing precision, recall, and F-score metrics for metagenomes of varying complexity. "Perfect" simulated reads have no errors and even abundances across organisms (A, D, G), "Uneven" have no errors and randomly varied abundances across organisms (B, E, H), and "True" have simulated errors specific to each read type and varied abundances across organisms (C, F, I). Significance was calculated using a Mann–Whitney U test with Bonferroni correction. Ns: non-significant p-value ($p > 0.05$; blank means $p = 1$), *: $p \leq 0.05$, **: $p \leq 0.01$, ***: $p \leq 0.001$, ****: $p \leq 0.0001$.

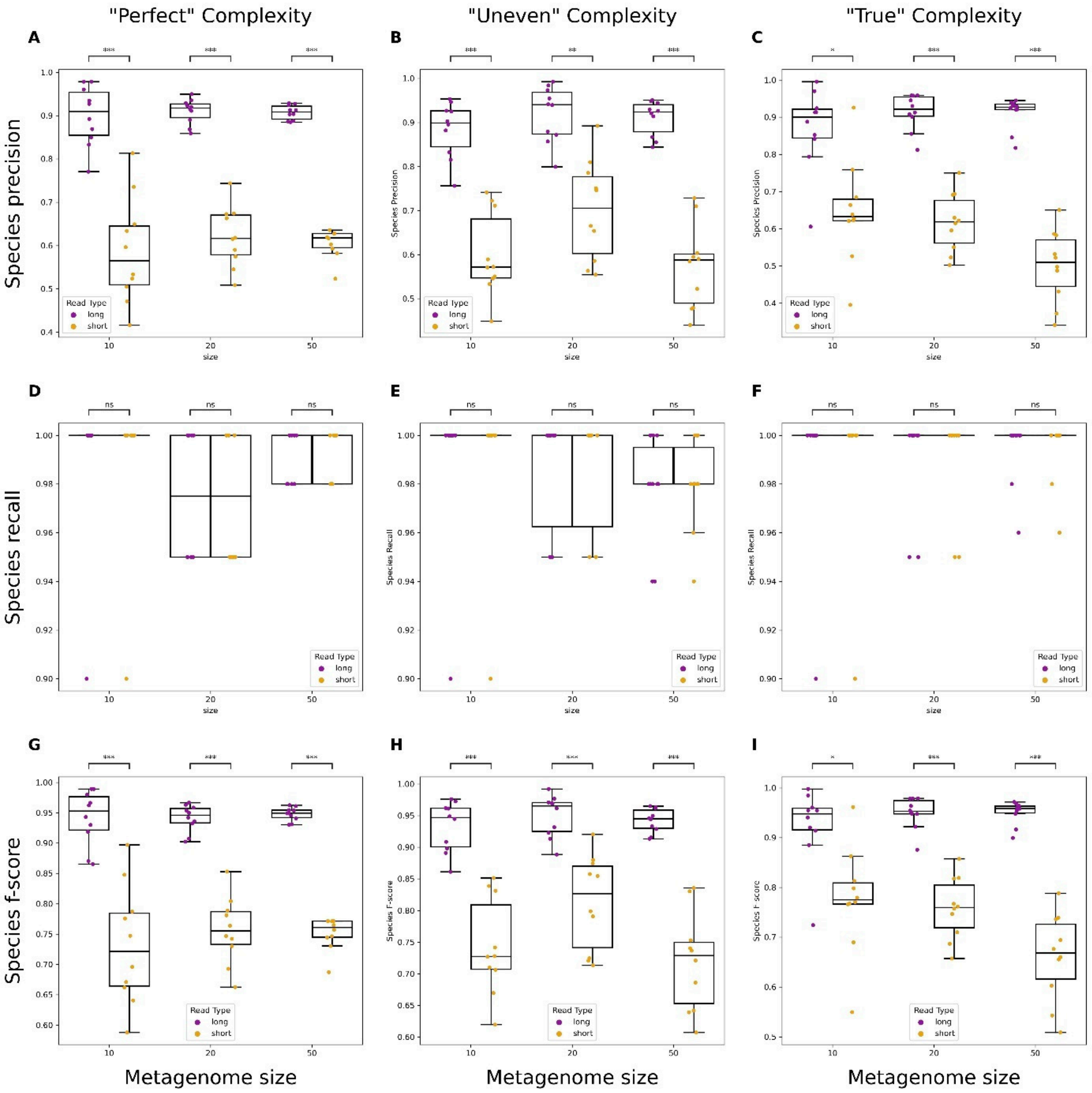

Supplemental Figure S3. Performance evaluation of species-level classification using short and long reads. Boxplots showing precision, recall, and F-score metrics for metagenomes of varying complexity. "Perfect" simulated reads have no errors and even abundances across organisms (A, D, G), "Uneven" have no errors and randomly varied abundances across organisms (B, E, H), and "True" have simulated errors specific to each read type and varied abundances across organisms (C, F, I). Significance was calculated using a Mann–Whitney U test with Bonferroni correction. Ns: non-significant p-value ($p > 0.05$; blank means $p = 1$), *: $p \leq 0.05$, **: $p \leq 0.01$, ***: $p \leq 0.001$, ****: $p \leq 0.0001$.

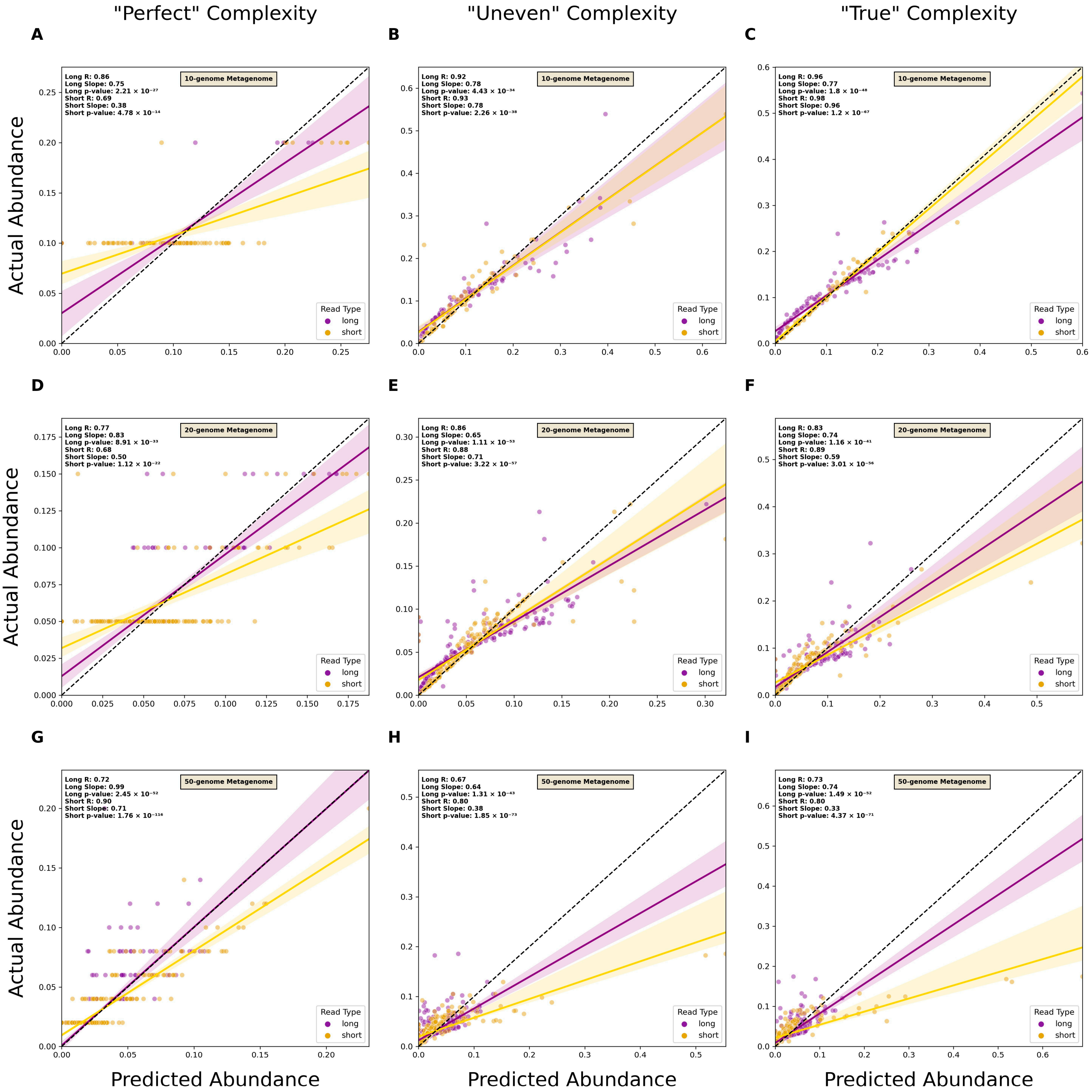

Figure S4. Comparison of short- and long-read capacity for genus-level relative abundance estimation. Scatterplots of predicted versus actual abundance values for genomes present in simulated metagenomes from short- and long-read data. Read types were compared across metagenomes of varying complexity at both the genus and species level. “Perfect” datasets consisted of reads without sequencing errors and organism abundance was evenly distributed (A, D, G), “Uneven” consisted of reads without sequencing errors and randomly distributed abundances of organisms (B, E, H), and “True” consisted of reads with simulated errors and randomly distributed abundances of organisms (C, F, I). A linear regression line was plotted for each read type. Each line has its reported R-value, slope, and p-value. The dotted line represents the 1-to-1 line where values of predicted abundance match values of actual abundance.

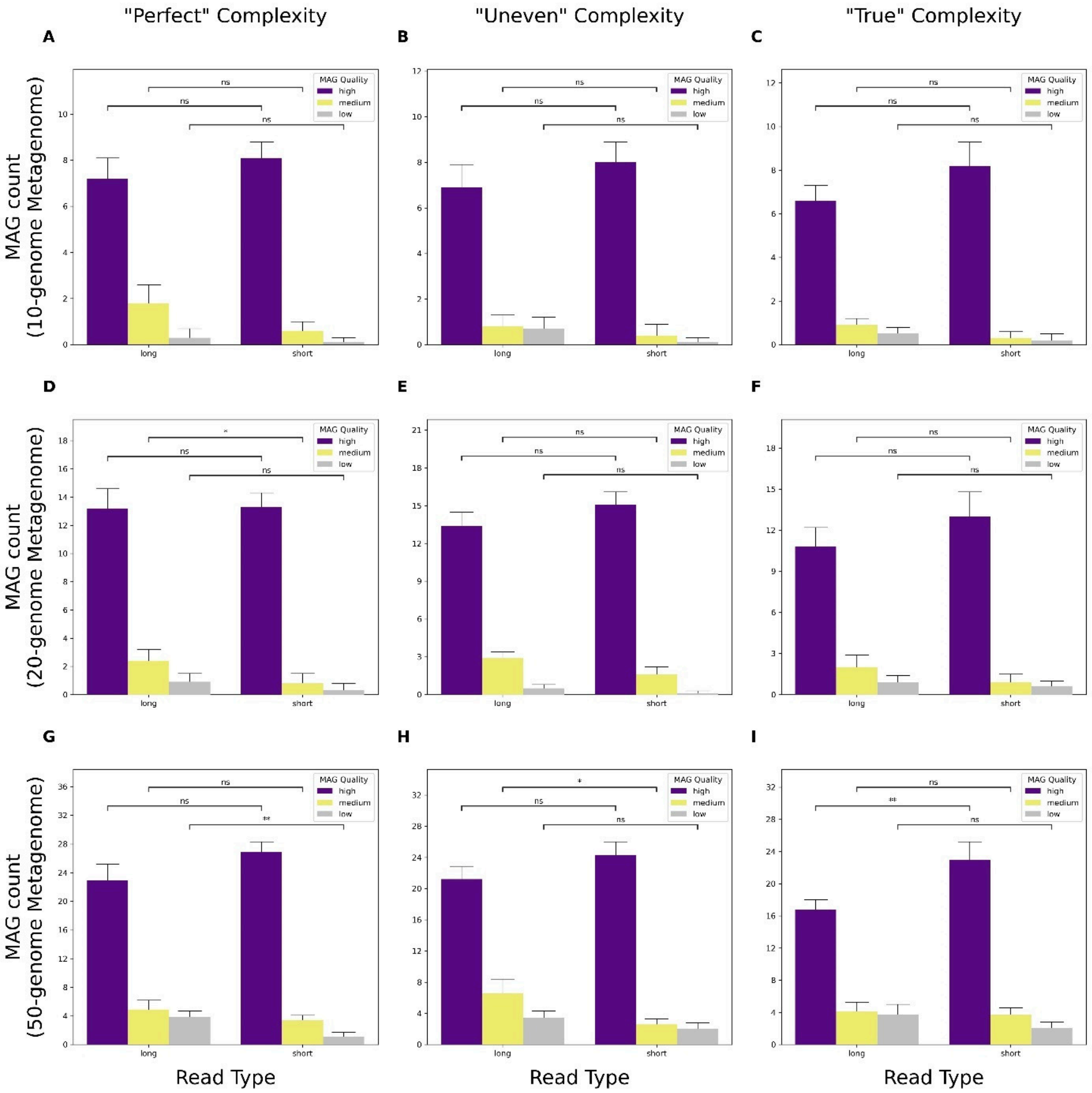

Supplemental Figure S5. Comparison of metagenome-assembled genome (MAG) counts recovered from short- and long-read assemblies by quality. Bar plots depict the total number of MAGs of a given quality recovered from both short- and long-read assemblies. "Perfect" denotes that simulated data have no errors and abundances are evenly distributed for each organism in the metagenome (S4A-C), "Uneven" have no errors, but variable abundances for each organism (S4D-F), and "True" have simulated sequencing errors based on the read type, as well as variable abundances for each organism (S4G-I). Significance was calculated using a Mann–Whitney U Test with Bonferroni correction. ns: non-significant p-value ($p > 0.05$), *: $p \leq 0.05$, **: $p \leq 0.01$, ***: $p \leq 0.001$, ****: $p \leq 0.0001$.

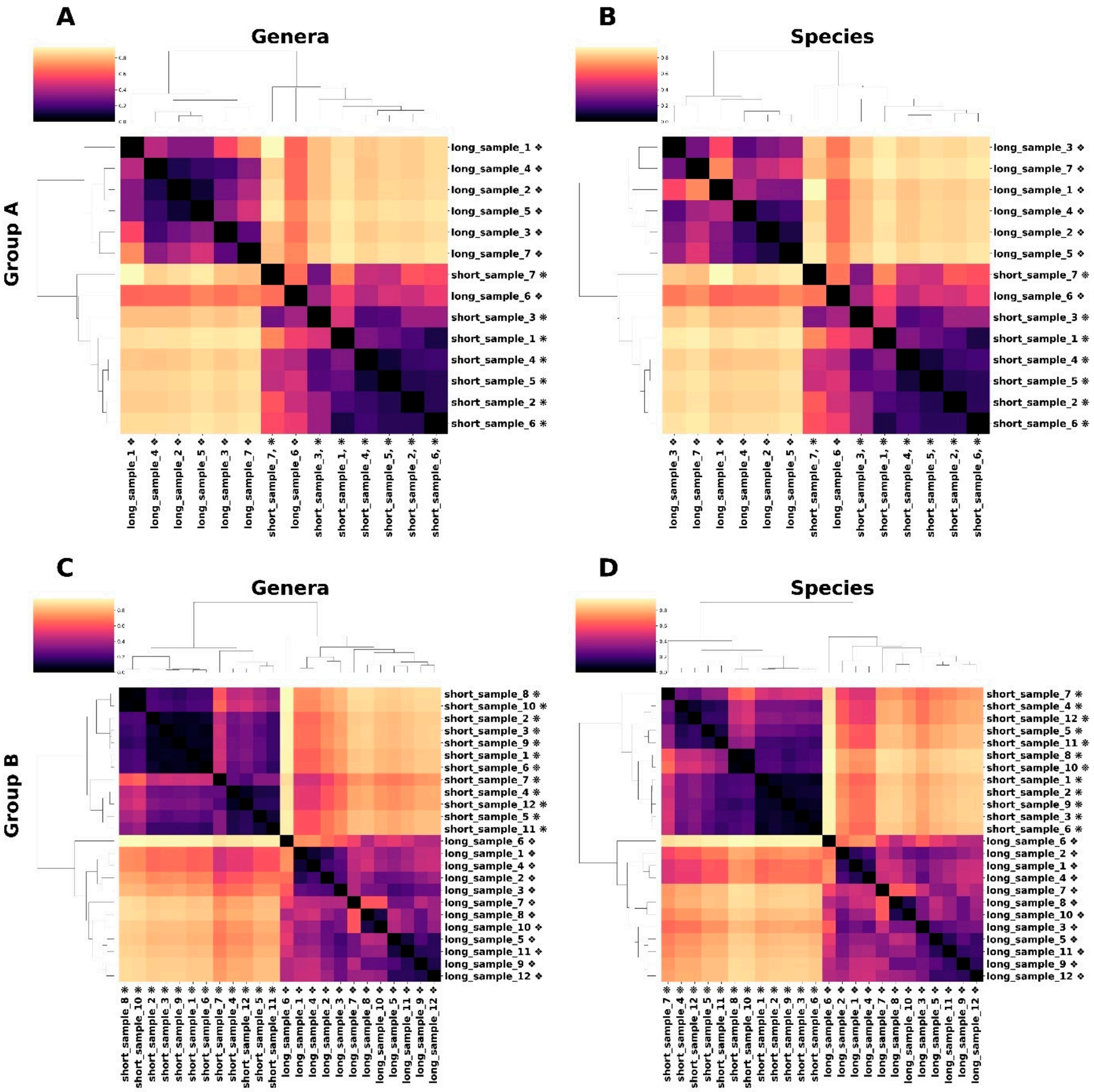

Supplemental Figure S6. Hierarchically clustered heatmap of Bray–Curtis Dissimilarity. Heatmap visualizing the dissimilarity of samples based on the read type used in beta-diversity assessment. Brighter values indicate lower similarity (or higher Bray–Curtis dissimilarity), darker values indicate higher similarity (or lower Bray–Curtis dissimilarity). ❖: Samples consist of long-read data. ❋: Samples consist of short-read data.

Table S1. Results of Mann–Whitney U testing for genome fraction, NGA50, and number of misassemblies.

simulated metagenome	complexity[1]	metaQUAST metric	statistic	p-value[2]
10-genome metagenome	perfect	genome fraction	7600.5	$1.79x10^{-10}$
10-genome metagenome	perfect	NGA50	8465	$2.56x10^{-17}$
10-genome metagenome	perfect	number of misassemblies	6473	$5.46x10^{-8}$
20-genome metagenome	perfect	genome fraction	27671.5	$3.04x10^{-11}$
20-genome metagenome	perfect	NGA50	30657.5	$3.03x10^{-20}$
20-genome metagenome	perfect	number of misassemblies	29583.5	$1.24x10^{-26}$
50-genome metagenome	perfect	genome fraction	134024	0.048
50-genome metagenome	perfect	NGA50	156277	$7.40x10^{-12}$
50-genome metagenome	perfect	number of misassemblies	194570	$3.23x10^{-67}$
10-genome metagenome	uneven	genome fraction	7433	$2.59x10^{-9}$
10-genome metagenome	uneven	NGA50	8152	$1.35x10^{-14}$
10-genome metagenome	uneven	number of misassemblies	6008	$4.41x10^{-3}$
20-genome metagenome	uneven	genome fraction	28562.5	$1.17x10^{-13}$
20-genome metagenome	uneven	NGA50	32254	$2.99x10^{-26}$
20-genome metagenome	uneven	number of misassemblies	20066	$9.49x10^{-1}$
50-genome metagenome	uneven	genome fraction	137225	$7.42x10^{-3}$
50-genome metagenome	uneven	NGA50	169597	$1.32x10^{-22}$
50-genome metagenome	uneven	number of misassemblies	133231	$6.46x10^{-2}$
10-genome metagenome	true	genome fraction	7812	$5.66x10^{-12}$
10-genome metagenome	true	NGA50	8341	$3.26x10^{-16}$
10-genome metagenome	true	number of misassemblies	5768.5	$1.98x10^{-3}$
20-genome metagenome	true	genome fraction	26882.5	$2.51x10^{-9}$
20-genome metagenome	true	NGA50	29641.5	$7.20x10^{-17}$
20-genome metagenome	true	number of misassemblies	20560	$5.84x10^{-1}$
50-genome metagenome	true	genome fraction	138988	$2.19x10^{-3}$
50-genome metagenome	true	NGA50	170176	$3.94x10^{-23}$
50-genome metagenome	true	number of misassemblies	122815	$6.20x10^{-1}$

[1] "Perfect" indicates that reads had no errors and abundances were evenly distributed. "Uneven" denotes that reads had no errors but variable abundances for each organism. "True" reads had simulated sequencing errors based on the read type and variable abundances for the organisms.
[2]P-value from Mann–Whitney U test with Bonferroni correction.

Table S2. Bin and metagenome-assembled genome (MAG) counts from metagenomic samples. Total bins recovered for each metagenomic sample and counts of MAGs identified from those bins. MAGs were further separated by quality as described in the methods section.

sample[1]	metagenome set[2]	bin count (long)[3a]	bin count (short)[3b]	mag count (long)	mag count (short)	high (long)	high (short)	medium (long)	medium (short)	low (long)	low (short)
mock_metagenome_1	10_genomes_perfect	19	12	10	10	8	10	2	0	0	0
mock_metagenome_2	10_genomes_perfect	11	11	9	9	6	8	3	0	0	1
mock_metagenome_3	10_genomes_perfect	10	11	10	10	10	10	0	0	0	0
mock_metagenome_4	10_genomes_perfect	12	10	9	9	7	7	1	2	1	0
mock_metagenome_5	10_genomes_perfect	17	10	9	7	4	6	5	1	0	0
mock_metagenome_6	10_genomes_perfect	17	9	8	8	7	8	1	0	0	0
mock_metagenome_7	10_genomes_perfect	12	10	10	8	8	8	2	0	0	0
mock_metagenome_8	10_genomes_perfect	9	10	9	9	8	8	1	1	0	0
mock_metagenome_9	10_genomes_perfect	14	11	9	9	6	8	1	1	2	0
mock_metagenome_10	10_genomes_perfect	16	13	10	9	8	8	2	1	0	0
mock_metagenome_1	10_genomes_true	11	11	9	10	8	9	0	1	1	0
mock_metagenome_2	10_genomes_true	11	11	9	10	8	10	1	0	0	0
mock_metagenome_3	10_genomes_true	11	8	7	8	6	8	1	0	0	0
mock_metagenome_4	10_genomes_true	12	12	9	9	8	9	0	0	1	0
mock_metagenome_5	10_genomes_true	11	8	7	8	6	8	1	0	0	0
mock_metagenome_6	10_genomes_true	12	12	7	9	6	7	1	1	0	1
mock_metagenome_7	10_genomes_true	13	10	7	8	5	8	2	0	0	0
mock_metagenome_8	10_genomes_true	15	10	9	10	7	10	1	0	1	0
mock_metagenome_9	10_genomes_true	9	9	7	5	5	3	1	1	1	1
mock_metagenome_10	10_genomes_true	14	10	9	10	7	10	1	0	1	0
mock_metagenome_1	10_genomes_uneven	13	10	9	9	8	9	1	0	0	0
mock_metagenome_2	10_genomes_uneven	15	9	9	8	6	7	2	1	1	0
mock_metagenome_3	10_genomes_uneven	10	11	9	9	8	9	1	0	0	0
mock_metagenome_4	10_genomes_uneven	10	12	9	10	8	10	1	0	0	0
mock_metagenome_5	10_genomes_uneven	12	9	8	7	7	7	0	0	1	0
mock_metagenome_6	10_genomes_uneven	16	17	7	6	6	6	0	0	1	0
mock_metagenome_7	10_genomes_uneven	18	10	7	9	3	9	2	0	2	0
mock_metagenome_8	10_genomes_uneven	15	13	9	9	6	7	1	2	2	0
mock_metagenome_9	10_genomes_uneven	9	9	7	8	7	6	0	1	0	1
mock_metagenome_10	10_genomes_uneven	10	10	10	10	10	10	0	0	0	0
mock_metagenome_1	20_genomes_perfect	26	18	18	13	14	13	4	0	0	0
mock_metagenome_2	20_genomes_perfect	22	18	17	15	14	14	0	1	3	0
mock_metagenome_3	20_genomes_perfect	24	19	17	14	13	13	3	1	1	0
mock_metagenome_4	20_genomes_perfect	19	20	18	17	16	15	2	0	0	2
mock_metagenome_5	20_genomes_perfect	22	21	19	17	17	16	1	1	1	0
mock_metagenome_6	20_genomes_perfect	26	18	14	13	10	12	2	1	2	0
mock_metagenome_7	20_genomes_perfect	33	32	11	13	10	10	1	3	0	0
mock_metagenome_8	20_genomes_perfect	22	22	14	13	10	12	3	0	1	1
mock_metagenome_9	20_genomes_perfect	27	17	19	13	14	13	4	0	1	0
mock_metagenome_10	20_genomes_perfect	22	21	18	16	14	15	4	1	0	0
mock_metagenome_1	20_genomes_true	17	20	11	14	11	12	0	1	0	1
mock_metagenome_2	20_genomes_true	24	17	14	15	12	15	1	0	1	0
mock_metagenome_3	20_genomes_true	23	23	14	17	12	13	1	2	1	2
mock_metagenome_4	20_genomes_true	24	19	16	18	12	15	3	2	1	1
mock_metagenome_5	20_genomes_true	24	20	14	14	14	12	0	2	0	0
mock_metagenome_6	20_genomes_true	26	20	14	13	7	12	5	0	2	1
mock_metagenome_7	20_genomes_true	15	21	9	9	7	7	2	2	0	0
mock_metagenome_8	20_genomes_true	19	21	12	12	9	12	3	0	0	0
mock_metagenome_9	20_genomes_true	23	22	14	14	10	13	2	0	2	1
mock_metagenome_10	20_genomes_true	21	20	19	19	14	19	3	0	2	0
mock_metagenome_1	20_genomes_uneven	26	18	18	16	14	16	3	0	1	0
mock_metagenome_2	20_genomes_uneven	20	18	16	17	12	17	3	0	1	0
mock_metagenome_3	20_genomes_uneven	23	19	16	16	14	16	2	0	0	0
mock_metagenome_4	20_genomes_uneven	19	20	16	14	13	13	3	1	0	0
mock_metagenome_5	20_genomes_uneven	26	21	19	19	15	17	3	2	1	0
mock_metagenome_6	20_genomes_uneven	27	23	18	19	17	16	1	3	0	0
mock_metagenome_7	20_genomes_uneven	23	20	16	16	13	14	3	2	0	0
mock_metagenome_8	20_genomes_uneven	22	21	19	20	14	16	4	3	1	1
mock_metagenome_9	20_genomes_uneven	25	22	15	16	10	13	4	3	1	0
mock_metagenome_10	20_genomes_uneven	20	19	15	15	12	13	3	2	0	0
mock_metagenome_1	50_genomes_perfect	57	49	33	30	19	22	9	5	5	3
mock_metagenome_2	50_genomes_perfect	47	48	35	36	28	31	6	4	1	1
mock_metagenome_3	50_genomes_perfect	51	43	32	32	22	28	5	3	5	1
mock_metagenome_4	50_genomes_perfect	50	50	29	31	19	27	6	2	4	2
mock_metagenome_5	50_genomes_perfect	56	41	29	33	21	29	3	3	5	1
mock_metagenome_6	50_genomes_perfect	47	46	33	32	23	28	5	4	5	0
mock_metagenome_7	50_genomes_perfect	48	57	33	30	24	27	4	2	5	1
mock_metagenome_8	50_genomes_perfect	49	44	26	30	17	25	6	5	3	0
mock_metagenome_9	50_genomes_perfect	44	44	34	33	28	28	4	3	2	2
mock_metagenome_10	50_genomes_perfect	54	43	33	27	28	24	1	3	4	0
mock_metagenome_1	50_genomes_true	52	39	26	33	16	28	4	3	6	2
mock_metagenome_2	50_genomes_true	50	38	28	30	19	24	4	4	5	2
mock_metagenome_3	50_genomes_true	52	38	25	29	18	23	3	5	4	1
mock_metagenome_4	50_genomes_true	56	40	26	32	14	27	5	3	7	2
mock_metagenome_5	50_genomes_true	50	37	23	24	16	19	2	4	5	1
mock_metagenome_6	50_genomes_true	51	42	22	28	18	18	4	7	0	3
mock_metagenome_7	50_genomes_true	52	41	24	31	18	26	3	1	3	4
mock_metagenome_8	50_genomes_true	41	42	24	28	16	20	6	4	2	4
mock_metagenome_9	50_genomes_true	39	42	25	23	13	18	8	4	4	1
mock_metagenome_10	50_genomes_true	46	35	23	30	20	27	2	2	1	1
mock_metagenome_1	50_genomes_uneven	49	38	31	32	22	27	6	3	3	2
mock_metagenome_2	50_genomes_uneven	44	32	28	27	19	24	6	2	3	1
mock_metagenome_3	50_genomes_uneven	47	43	33	28	20	21	8	4	5	3
mock_metagenome_4	50_genomes_uneven	52	37	33	27	22	26	10	0	1	1
mock_metagenome_5	50_genomes_uneven	39	32	26	23	22	20	2	2	2	1
mock_metagenome_6	50_genomes_uneven	56	39	32	29	16	24	12	3	4	2
mock_metagenome_7	50_genomes_uneven	48	37	31	28	23	22	5	4	3	2
mock_metagenome_8	50_genomes_uneven	53	39	32	30	19	28	7	2	6	0
mock_metagenome_9	50_genomes_uneven	48	41	28	29	23	22	3	3	2	4
mock_metagenome_10	50_genomes_uneven	61	47	38	36	26	29	7	3	5	4

[1]Samples consist of the 10 metagenomes simulated for a given complexity. [2]A metagenome set is the given complexity level for a collection of simulated metagenomes. [3a]Sum of all bins predicted by MetaBAT2 from long-read assemblies. [3b]Sum of all bins predicted by MetaBAT2 from short-read assemblies.
