# Supplementary figures and images for: Improving Bacterial Metagenomic Research through Long-Read Sequencing
Source: Microorganisms. 2024 May 4;12(5):935. doi: 10.3390/microorganisms12050935 (PMC11124196; doi:10.3390/microorganisms12050935)

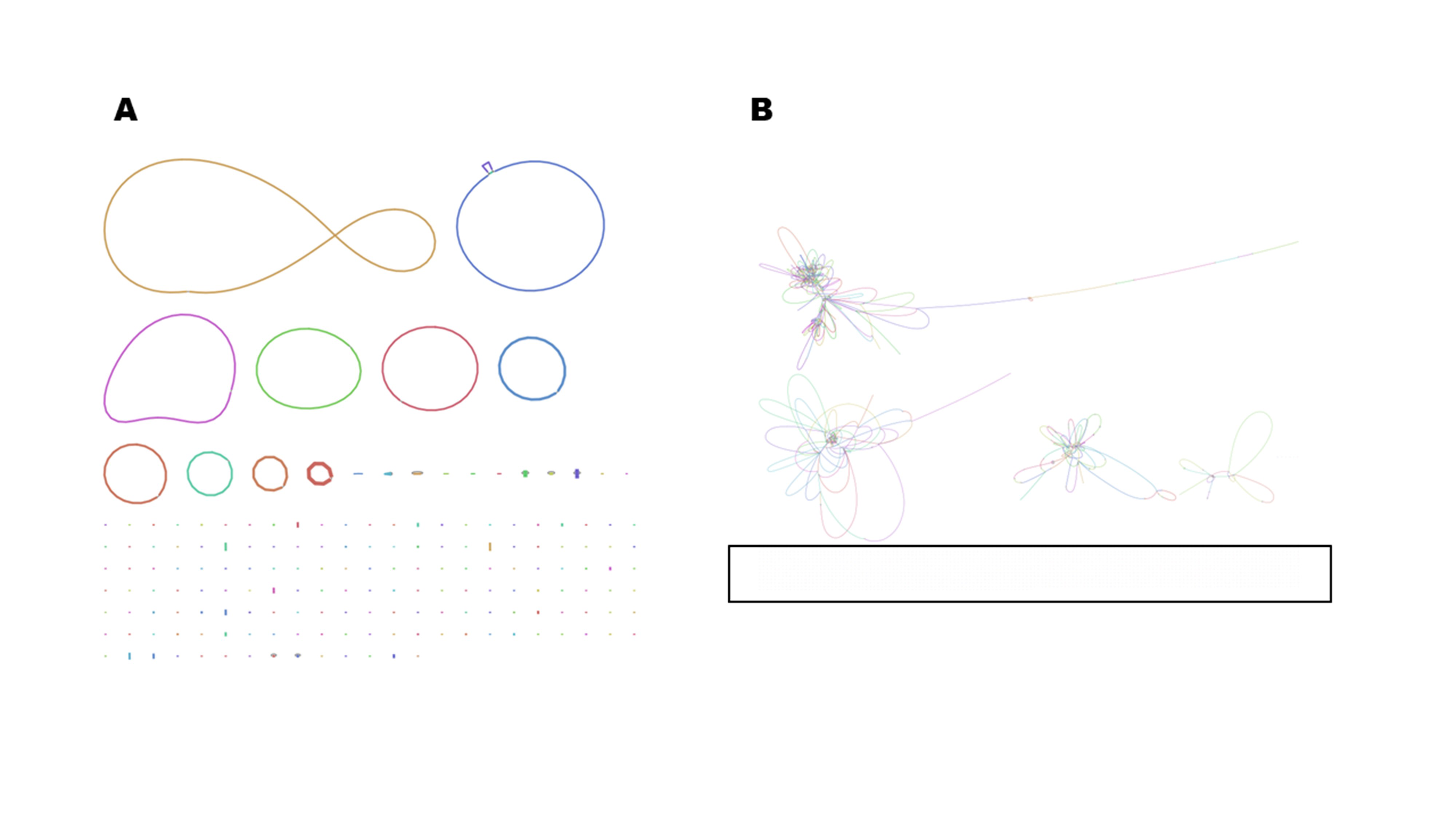

Supplement: Supplementary file 1 [file microorganisms-12-00935-s001.zip › Figure_S1_bandage-comparison-graphs.png]

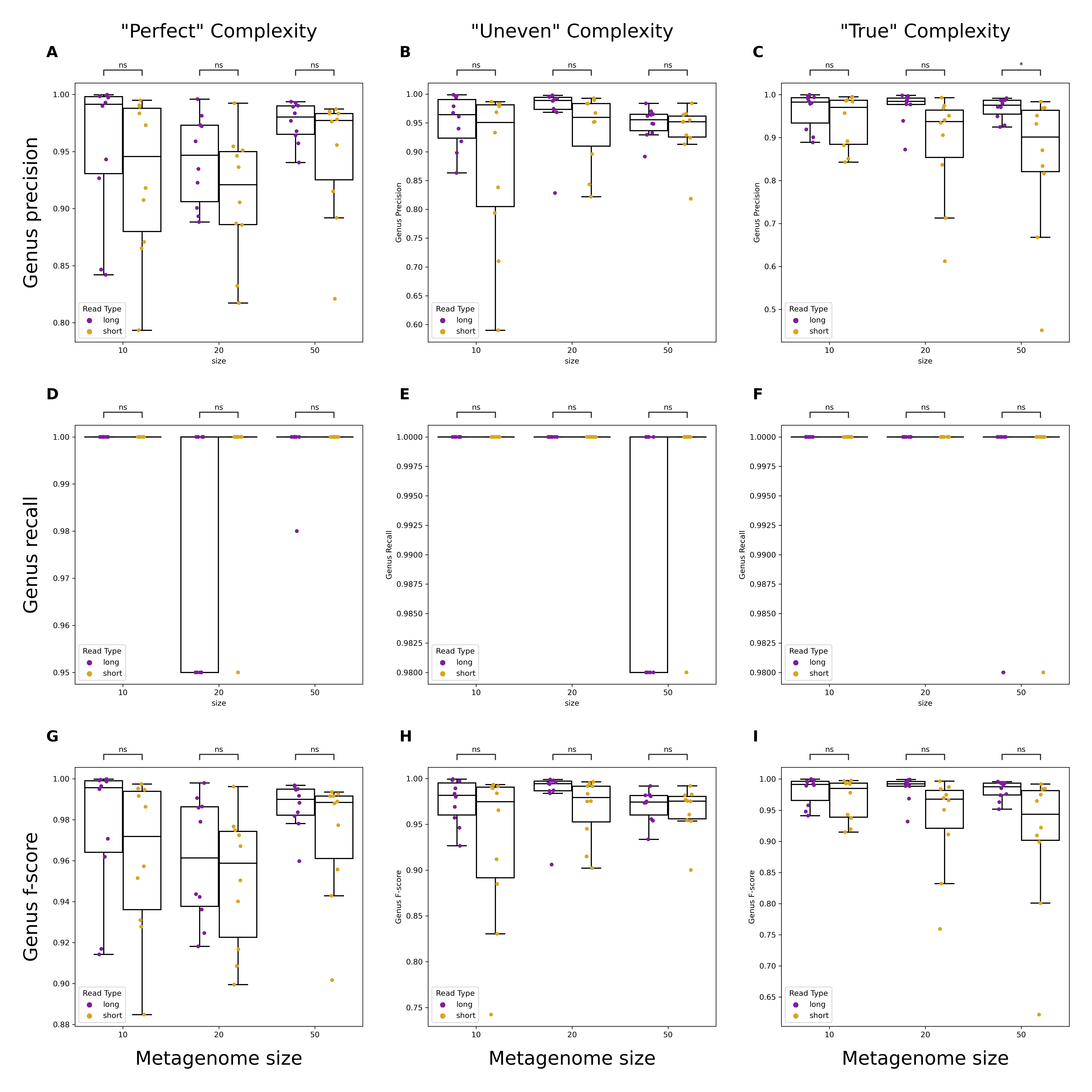

Supplement: Supplementary file 1 [file microorganisms-12-00935-s001.zip › Figure_S2_reads_genus_level_performance_figure_two-sided.png]

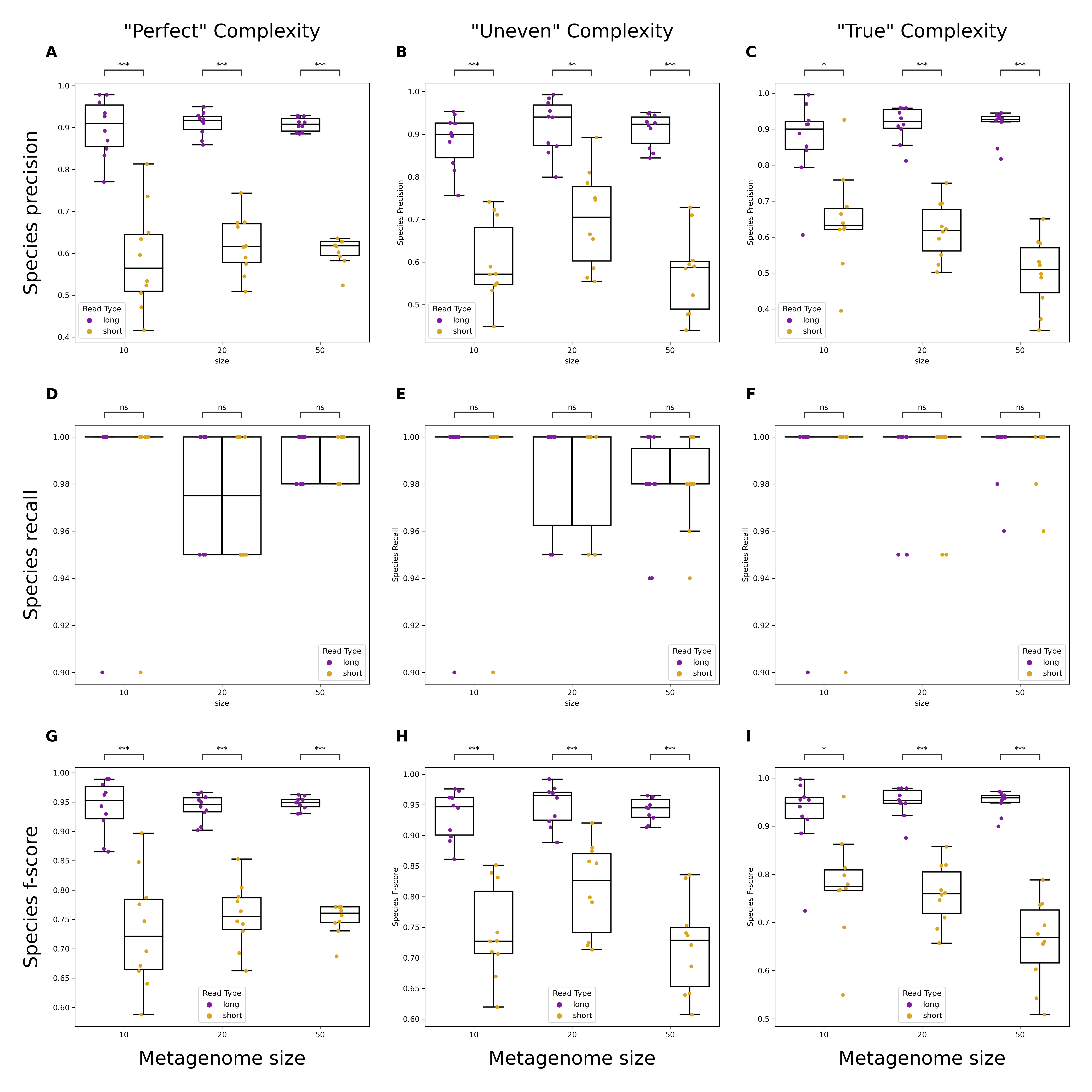

Supplement: Supplementary file 1 [file microorganisms-12-00935-s001.zip › Figure_S3_reads_species_level_performance_figure_two-sided.png]

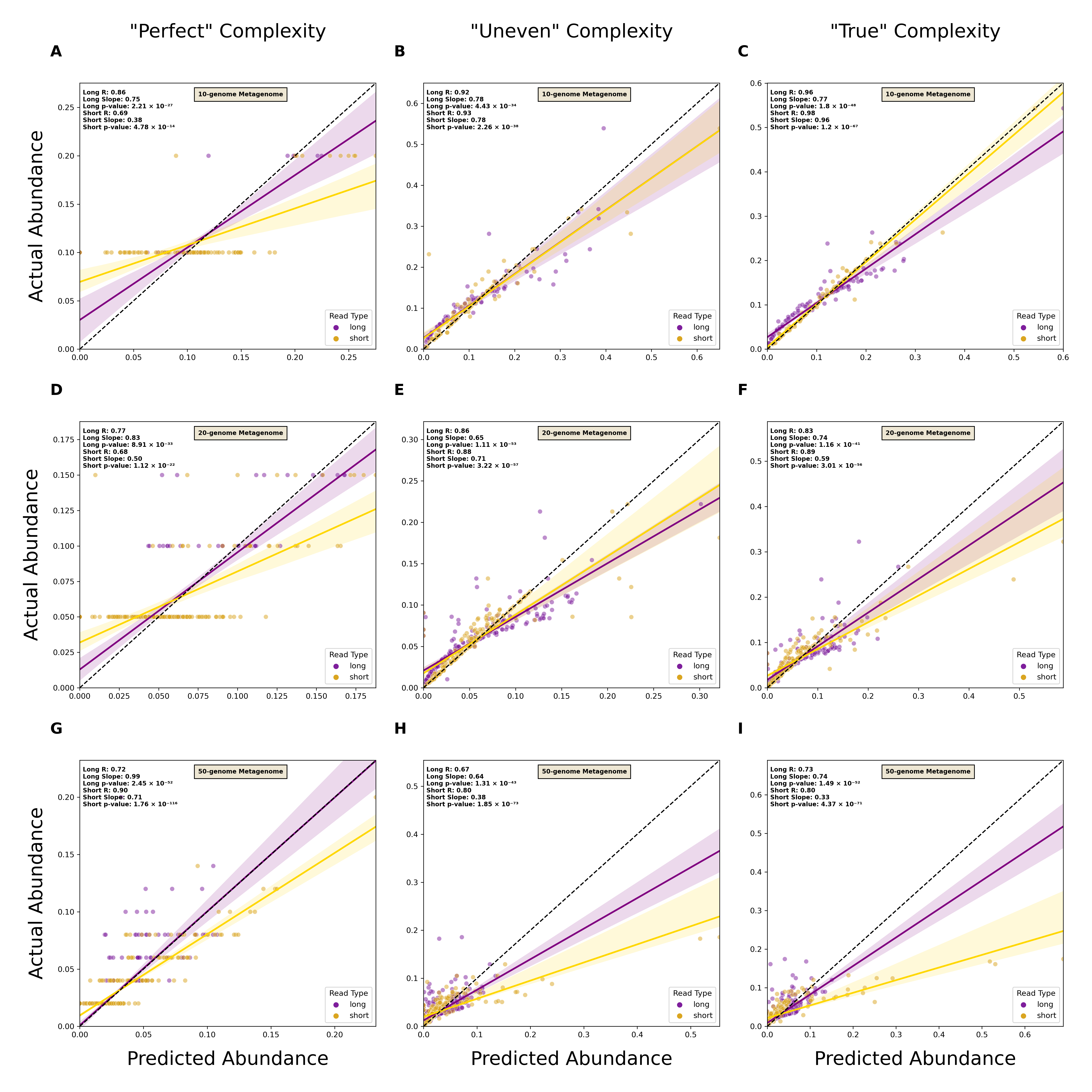

Supplement: Supplementary file 1 [file microorganisms-12-00935-s001.zip › Figure_S4_Genus_scatterplots.png]

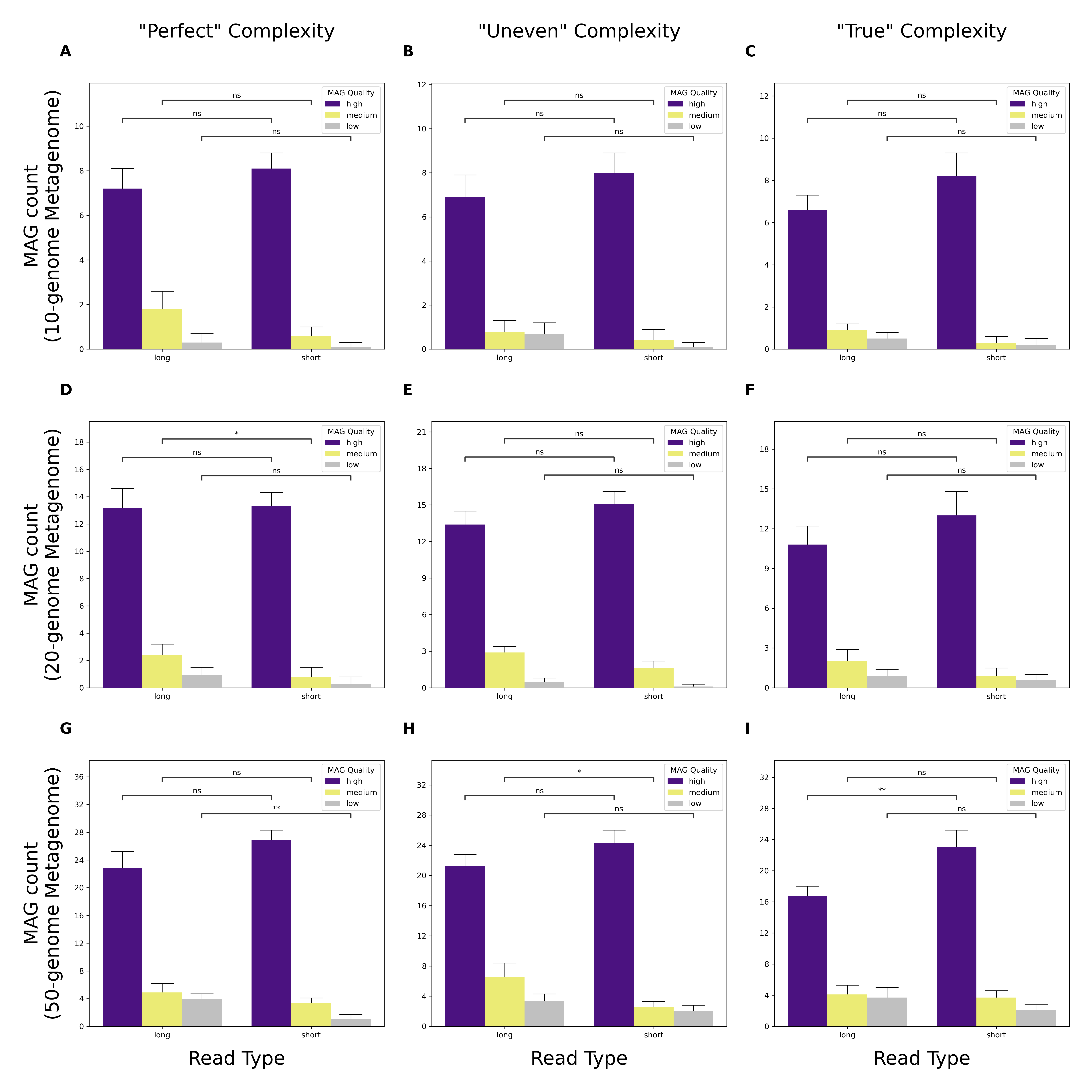

Supplement: Supplementary file 1 [file microorganisms-12-00935-s001.zip › Figure_S5_total_quality_MAG_recovery_counts_two-sided.png]

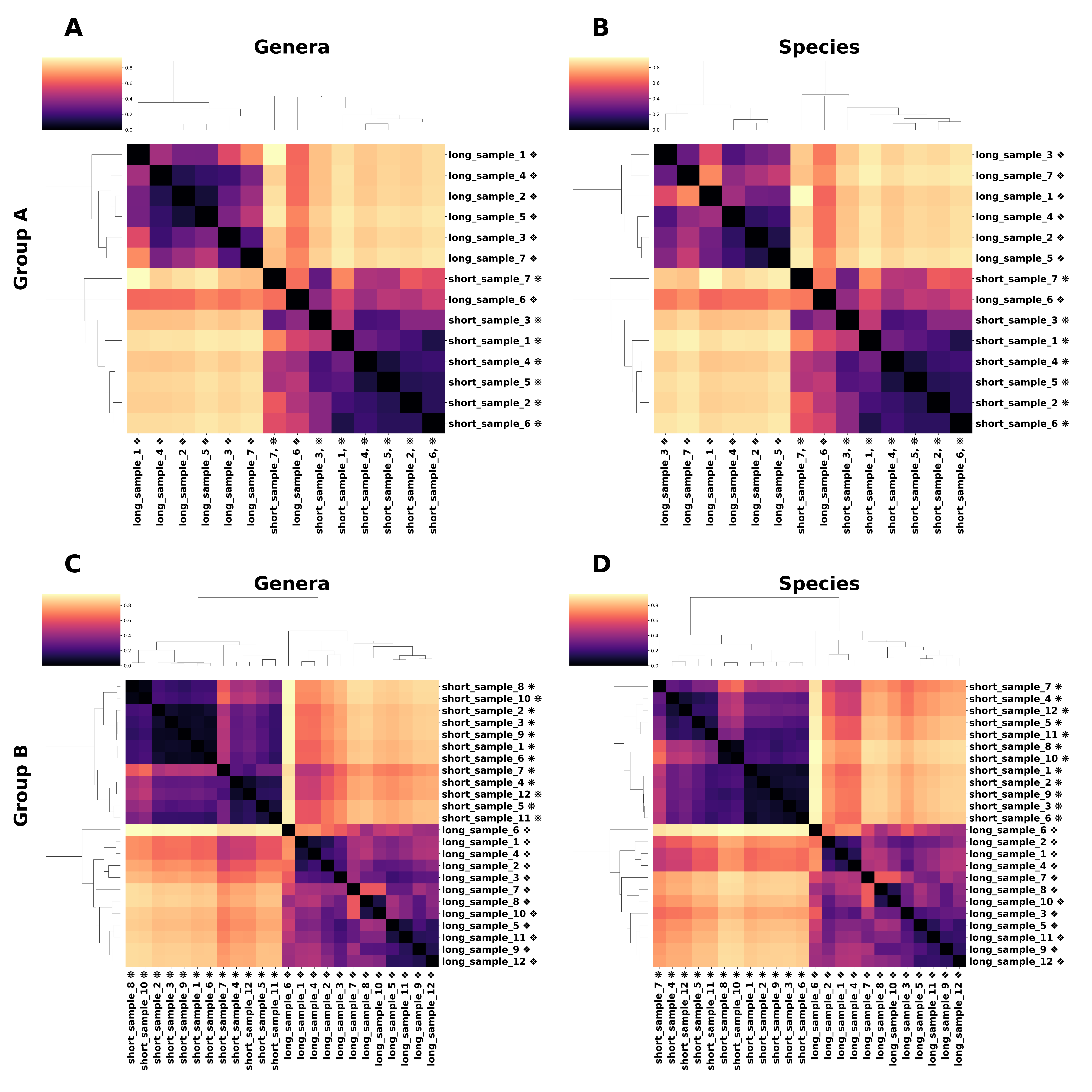

Supplement: Supplementary file 1 [file microorganisms-12-00935-s001.zip › Figure_S6_taxa-classification-comparison-clustermap.png]
